# Crystal structure and Hirshfeld surface analysis of (*E*)-1-[2,2-di­chloro-1-(4-nitro­phen­yl)ethen­yl]-2-(4-fluoro­phen­yl)diazene

**DOI:** 10.1107/S2056989019000707

**Published:** 2019-01-18

**Authors:** Zeliha Atioğlu, Mehmet Akkurt, Namiq Q. Shikhaliyev, Gulnar T. Suleymanova, Khanim N. Bagirova, Flavien A. A. Toze

**Affiliations:** a İlke Education and Health Foundation, Cappadocia University, Cappadocia Vocational College, The Medical Imaging Techniques Program, 50420 Mustafapaşa, Ürgüp, Nevşehir, Turkey; b Department of Physics, Faculty of Sciences, Erciyes University, 38039 Kayseri, Turkey; c Organic Chemistry Department, Baku State University, Z. Xalilov str. 23, Az, 1148 Baku, Azerbaijan; d Department of Chemistry, Faculty of Sciences, University of Douala, PO Box 24157, Douala, Republic of Cameroon

**Keywords:** crystal structure, 4-fluoro­phenyl ring, nitro-substituted benzene ring, hydrogen bonding, Hirshfeld surface analysis

## Abstract

In the title compound, C_14_H_8_Cl_2_FN_3_O_2_, the 4-fluoro­phenyl ring and the nitro-substituted benzene ring form a dihedral angle of 63.29 (8)°. In the crystal, mol­ecules are linked by C—H⋯O hydrogen bonds into chains running parallel to the *c* axis. The crystal packing is further stabilized by C—Cl⋯π, C—F⋯π and N—O⋯π inter­actions. The Hirshfeld surface analysis of the crystal structure indicates that the most important contributions to the crystal packing are from H⋯O/O⋯H (15.5%), H⋯H (15.3%), Cl⋯H/H⋯Cl (13.8%), C⋯H/H⋯C (9.5%) and F⋯H/H⋯F (8.2%) inter­actions.

## Abstract 2

The dihedral angle between the 4-fluoro­phenyl ring and the nitro-substituted benzene ring of the title compound is 63.29 (8)°. In the crystal, mol­ecules are linked by C—H⋯O hydrogen bonds into chains parallel to the *c* axis. The crystal packing is further stabilized by C—Cl⋯π, C—F⋯π and N—O⋯π inter­actions

## Chemical context   

Non-covalent inter­actions, such as hydrogen, aerogen, halogen, chalcogen, pnicogen, tetrel and icosa­gen bonds, as well as *n*–π*, *π–π* stacking, *π*–cation, *π*–anion and hydro­phobic inter­actions, can control or organize the conformation, aggregation, tertiary and quaternary structures of the mol­ecule, its stabilization and particular properties (Akbari Afkhami *et al.*, 2017 ▸; Desiraju, 1995 ▸; Gurbanov *et al.*, 2018 ▸; Hazra *et al.*, 2018 ▸; Jlassi *et al.*, 2014 ▸; Kvyatkovskaya *et al.*, 2017 ▸; Legon, 2017 ▸, Maharramov *et al.*, 2009 ▸, 2018 ▸; Mahmoudi *et al.*, 2018*a* ▸,*b* ▸,*c* ▸; Mahmudov *et al.*, 2014 ▸, 2017 ▸; Mahmudov & Pombeiro, 2016 ▸; Scheiner 2013 ▸; Shikhaliyev *et al.*, 2013 ▸, 2018 ▸). On the other hand, azo dyes and related hydrazone ligands and their complexes have attracted attention over the past decades because of their potential biological, pharmacological and analytical applications (Borisova *et al.*, 2018 ▸; Gadzhieva *et al.*, 2006 ▸; Gurbanov *et al.*, 2017 ▸; Shetnev & Zubkov, 2017 ▸). Herein we report the structure and non-covalent inter­actions of the title compound.
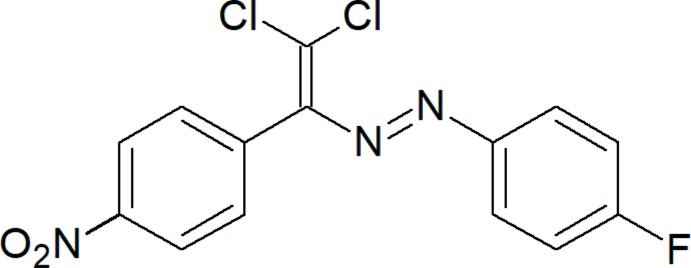


## Structural commentary   

**Figure 1:**
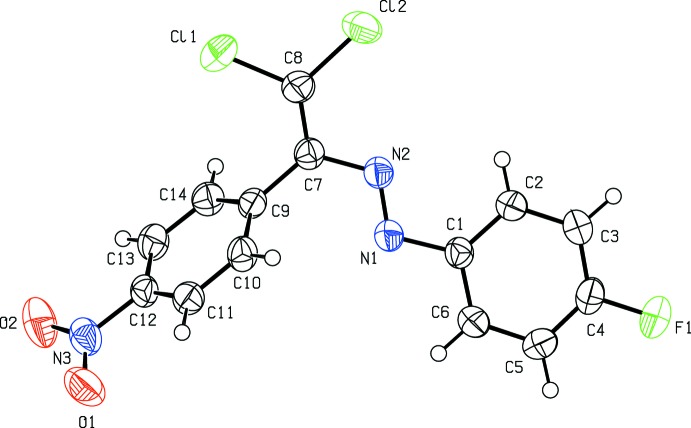
The mol­ecular structure of the title compound with displacement ellipsoids drawn at the 50% probability level.

The mol­ecular conformation of the title compound (Fig. 1 ▸) is not planar, the 4-fluoro­phenyl ring and the nitro-substituted benzene ring forming a dihedral angle of 63.29 (8)°. The C2—C1—N1—N2, C1—N1—N2—C7, N1—N2—C7—C8, N2—C7—C8—Cl1, N2—C7—C8—Cl2, Cl1—C8—C7—C9 and C8—C7—C9—C14 torsion angles are −1.1 (2), 178.86 (13), 174.62 (14), −176.19 (11), 2.9 (2), 5.1 (2) and 63.4 (2)°, respectively. Bond lengths (Allen *et al.*, 1987 ▸) and angles are within normal ranges and are comparable to those observed in related structures, *viz:* (2*E*)-1-(2-hy­droxy-5-methyl­phen­yl)-3-(4-meth­oxy­phen­yl)prop-2-en-1-one (Fun *et al.*, 2011*a* ▸), (2*E*)-3-(3-benzyl­oxyphen­yl)-1-(2-hy­droxy-5-methyl­phen­yl)prop-2-en-1-one (Fun *et al.*, 2011*b* ▸), (2*E*)-3-[3-(benz­yloxy)phen­yl]-1-(2-hy­droxy­phen­yl)prop-2-en-1-one (Fun *et al.*, 2011*c* ▸), (2*E*)-1-(2,5-di­meth­oxy­phen­yl)-3-(3-nitro­phen­yl)prop-2-en-1-one (Fun *et al.*, 2011*d* ▸) and (2*E*)-3-(3-nitro­phen­yl)-1-[4-(piperidin- 1-yl)phen­yl]prop-2-en-1-one (Fun *et al.*, 2012 ▸).

## Supra­molecular features and Hirshfeld surface analysis   

**Figure 2:**
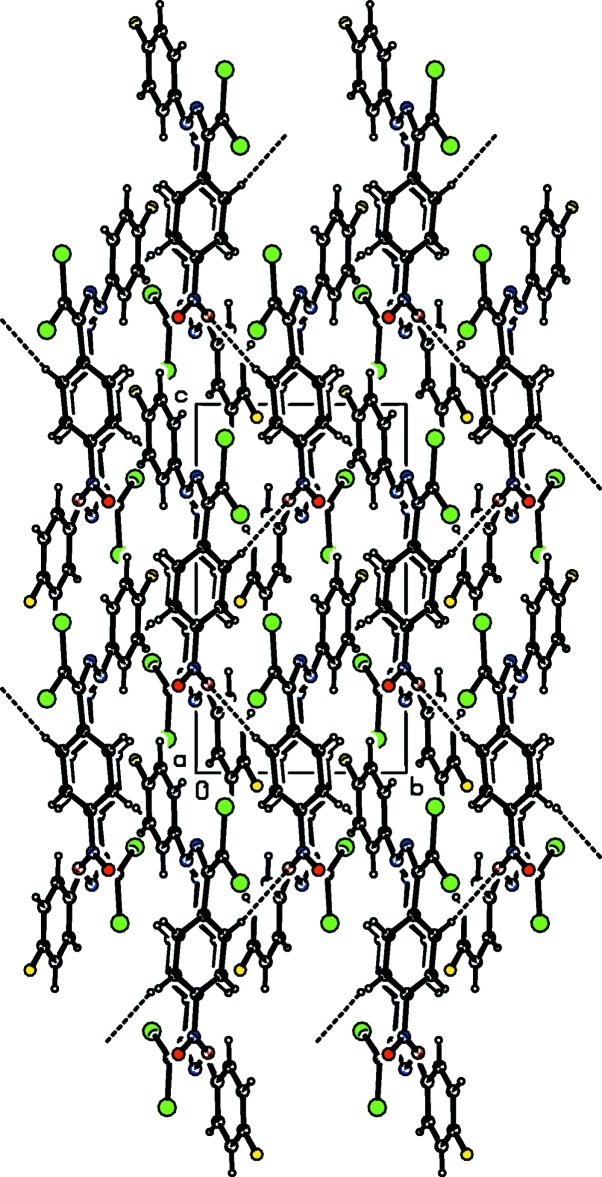
Crystal packing of the title compound, viewed down the *a* axis, showing the formation of chains parallel to the *c* axis through C—H⋯O hydrogen bonds (dashed lines).

In the crystal, mol­ecules are linked by C—H⋯O hydrogen bonds into chains parallel to the *c* axis (Table 1 ▸; Fig. 2 ▸). The crystal packing is further stabilized by weak C—Cl⋯π [Cl⋯*Cg*2(*x*, 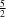 − *y*, 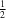 + *z*) = 3.6792 (8) Å], C—F⋯π [F⋯*Cg*1(1 − *x*, 2 − *y*, 2 − *z*) = 3.5408 (16) Å] and N—O⋯π inter­actions [O⋯*Cg*1(*x*, 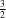 − *y*, −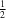 + *z*) = 3.9815 (16) Å] where *Cg*1 and *Cg*2 are the centroids of the C1–C6 and C9–C14 rings, respectively.

**Figure 3:**
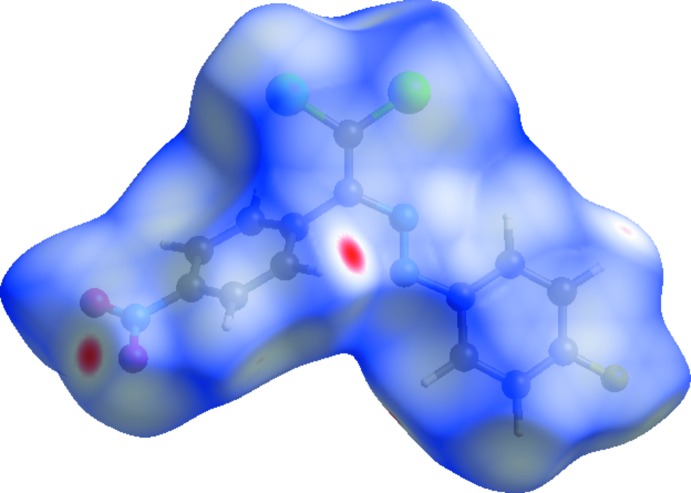
View of the three-dimensional Hirshfeld surface of the title compound plotted over *d*_norm_ in the range −0.1603 to 1.2420 a.u.

Hirshfeld surfaces and fingerprint plots were generated for the title compound using *CrystalExplorer* (McKinnon *et al.*, 2007 ▸) to qu­antify and visualize the inter­molecular inter­actions and to explain the observed crystal packing. The Hirshfeld surface mapped over *d*_norm_ using a standard surface resolution with a fixed colour scale of −0.1603 (red) to 1.2420 (blue) a.u. is shown in Fig. 3 ▸. The dark-red spots on the *d*_norm_ surface arise as a result of short inter­atomic contacts (Table 2 ▸), while the other weaker inter­molecular inter­actions appear as light-red spots. The red points, which represent closer contacts and negative *d*_norm_ values on the surface, correspond to the C—H⋯O inter­actions.

**Figure 4:**
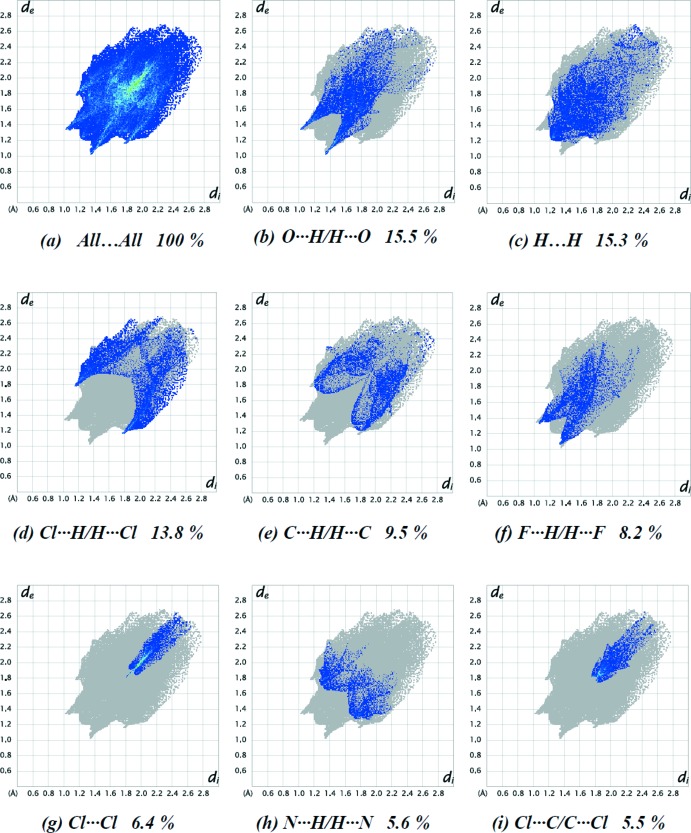
The full two-dimensional fingerprint plots for the title compound, showing (*a*) all inter­actions, and delineated into (*b*) O⋯H/H⋯O, (*c*) H⋯H, (*d*) Cl⋯H/H⋯Cl, (*e*) C⋯H/H⋯C, (*f*) F⋯H/H⋯F, (*g*) Cl⋯Cl, (*h*) N⋯H/H⋯N and (*i*) Cl⋯C/C⋯Cl inter­actions. The *d*_i_ and *d*_e_ values are the closest inter­nal and external distances (in Å) from given points on the Hirshfeld surface.

**Figure 5:**
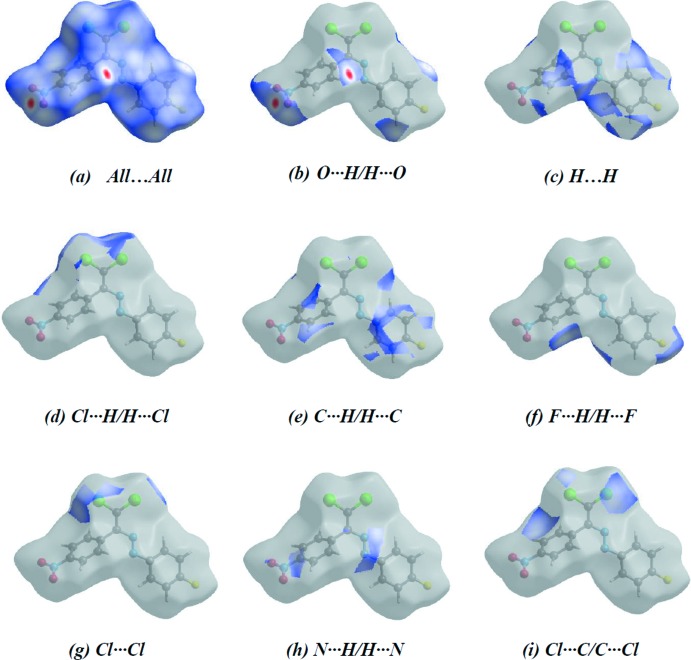
Hirshfeld surface representations with the function *d*_norm_ plotted onto the surface for (*a*) all inter­actions, (*b*) O⋯H/H⋯O, (*c*) H⋯H, (*d*) Cl⋯H/H⋯Cl, (*e*) C⋯H/H⋯C, (*f*) F⋯H/H⋯F, (*g*) Cl⋯Cl, (*h*) N⋯H/H⋯N and (*i*) Cl⋯C/C⋯Cl inter­actions.

**Figure 6:**
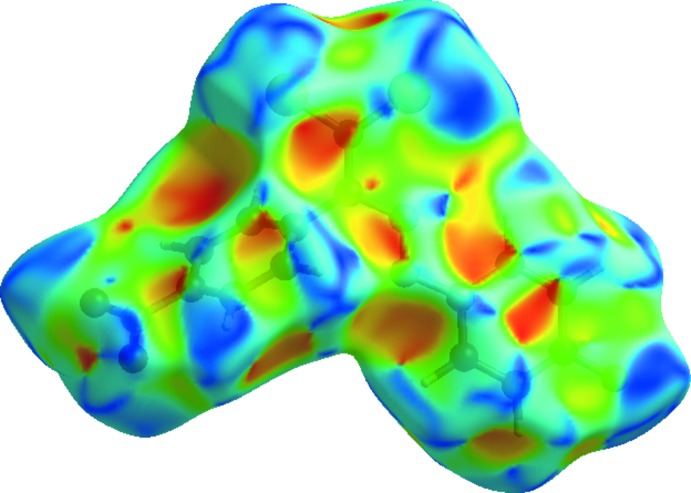
Hirshfeld surface of the title compound plotted over shape-index.

The percentage contributions of various contacts to the total Hirshfeld surface are shown in the two-dimensional fingerprint plots in Fig. 4 ▸. The reciprocal O⋯H/H⋯O inter­actions appear as two symmetrical broad wings with *d*_e_ + *d*_i_ ≃ 2.2 Å and contribute 15.5% to the Hirshfeld surface (Fig. 5 ▸ *b*). The reciprocal Cl⋯H/H⋯Cl, C⋯H/H⋯C and F⋯H/H⋯F inter­actions (13.8, 9.5 and 8.2% contributions, respectively) are present as sharp symmetrical spikes at diagonal axes *d*_e_ + *d*_i_ ≃ 2.9, 3.0 and 2.4 Å, respectively (Fig. 5 ▸ *d*–*f*). The small percentage contributions to the Hirshfeld surfaces from the various other inter­atomic contacts are listed in Table 3 ▸. Hirshfeld surface representations with the function *d*_norm_ plotted onto the surface for all inter­actions are shown in Fig. 5 ▸. The large number of O⋯H/H⋯O, H⋯H, Cl⋯H/H⋯Cl, C⋯H/H⋯C, F⋯H/H⋯F, Cl⋯Cl, N⋯H/H⋯N and Cl⋯C/C⋯Cl inter­actions suggest that van der Waals inter­actions and hydrogen bonding play a major role in the crystal packing (Hathwar *et al.*, 2015 ▸). The shape-index of the Hirshfeld surface is a tool for visualizing the π–π stacking by the presence of adjacent red and blue triangles; if there are no such triangles, then there are no π–π interactions. The plot of the Hirshfeld surface mapped over shape-index shown in Fig. 6 ▸ clearly suggests that there are no π–π interactions in the title compound.

## Synthesis and crystallization   

The title compound was synthesized according to the method reported by Shikhaliyev *et al.* (2018 ▸). A 20 mL screw-neck vial was charged with DMSO (10 mL), (*E*)-1-(4-fluoro­phen­yl)-2-(4-nitro­benzyl­idene)hydrazine (259 mg, 1 mmol), tetra­methyl­ethylenedi­amine (TMEDA; 295 mg, 2.5 mmol), CuCl (2 mg, 0.02 mmol) and CCl_4_ (20 mmol, 10 equiv). After 1–3 h (until TLC analysis showed complete consumption of the corresponding Schiff base), the reaction mixture was poured into a 0.01 *M* solution of HCl (100 mL, pH = 2–3), and extracted with di­chloro­methane (3 × 20 mL). The combined organic phase was washed with water (3 × 50 mL), brine (30 mL), dried over anhydrous Na_2_SO_4_ and concentrated *in vacuo* by rotary evaporator. The residue was purified by column chromatography on silica gel using appropriate mixtures of hexane and di­chloro­methane (3:1-1:1 *v*/*v*). Crystals suitable for X-ray analysis were obtained by slow evaporation of an ethanol solution. Yield (62%); m.p. 421 K. Analysis calculated for C_14_H_8_Cl_2_FN_3_O_2_ (*M =* 340.14): C, 49.44; H, 2.37; N, 12.35; found: C, 49.38; H, 2.40; N, 12.24%. ^1^H NMR (300 MHz, CDCl_3_) δ 8.32–8.29 (*d*, 2H, *J* = 9.21Hz), 7.81–7.77 (*m* 2H), 7.40–7.37 (*d*, 2H, *J* = 9.02Hz), 7.17–7.12 (*t*, 2H, *J* = 9.22Hz).^13^C NMR (75 MHz, CDCl_3_) δ 166.69, 163.32, 150.43, 149.17, 147.95, 139.40, 131.26, 125.51, 125.39, 123.41, 116.42, 116.11. ESI–MS: *m*/*z*: 341.06 [*M* + H]^+^.

## Refinement   

Crystal data, data collection and structure refinement details are summarized in Table 4 ▸. C-bound H atoms were constrained to an ideal geometry with C—H = 0.93 Å and refined as riding with *U*_iso_(H) = 1.2*U*_eq_(C). Three outliers (100, 110, 200) were omitted in the last cycles of refinement.

## Supplementary Material

Crystal structure: contains datablock(s) I. DOI: 10.1107/S2056989019000707/rz5248sup1.cif

Structure factors: contains datablock(s) I. DOI: 10.1107/S2056989019000707/rz5248Isup2.hkl

Supporting information file. DOI: 10.1107/S2056989019000707/rz5248Isup3.cml

CCDC reference: 1876976

Additional supporting information:  crystallographic information; 3D view; checkCIF report

## Figures and Tables

**Table 1:** Hydrogen-bond geometry (Å, °)

*D*—H⋯*A*	*D*—H	H⋯*A*	*D*⋯*A*	*D*—H⋯*A*
C10—H10⋯O1^i^	0.93	2.52	3.369 (2)	152

Symmetry code: (i) 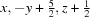.

**Table 2:** Summary of short inter­atomic contacts (Å) in the title compound

Contact	Distance	Symmetry operation
(C8) Cl1⋯C8 (Cl1)	3.6040 (16)	2 − *x*, 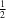 + *y*, 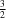 − *z*
(C13) H13⋯Cl1 (C8)	3.08	2 − *x*, 2 − *y*, 1 − *z*
(C8) Cl2⋯Cl2 (C8)	3.6506 (7)	2 − *x*, 2 − *y*, 2 − *z*
(C10) H10⋯O1 (N3)	2.52	*x*, 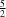 − *y*, 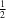 + *z*
(C4) F1⋯H11 (C11)	2.60	1 − *x*, − 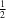 + *y*, 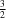 − *z*
(C4) F1⋯H6 (C6)	2.56	*x*, 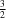 − *y*, 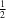 + *z*
(N3) O1⋯H3 (C3)	2.67	*x*, *y*, −1 + *z*
(C5) H5⋯O1 (N3)	2.74	1 − *x*, 2 − *y*, 1 − *z*
(N3) O1⋯H10 (C10)	2.52	*x*, 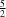 − *y*, − 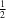 + *z*
(F1) C4⋯C4 (F1)	3.541 (3)	1 − *x*, 2 − *y*, 2 − *z*

**Table 3:** Percentage contributions of inter­atomic contacts to the Hirshfeld surface for the title compound

Contact	Percentage contribution
O⋯H/H⋯O	15.5
H⋯H	15.3
Cl⋯H/H⋯Cl	13.8
C⋯H/H⋯C	9.5
F⋯H/H⋯F	8.2
Cl⋯Cl	6.4
N⋯H/H⋯N	5.6
Cl⋯C/C⋯Cl	5.5
C⋯C	4.1
O⋯C/C⋯O	3.7
Cl⋯O/O⋯Cl	3.1
F⋯C/C⋯F	3.1
N⋯C/C⋯N	2.2
O⋯N/N⋯O	2.1
F⋯F	0.9
N⋯N	0.8

**Table 4:** Experimental details

Crystal data	
Chemical formula	C_14_H_8_Cl_2_FN_3_O_2_
*M*_r_	340.13
Crystal system, space group	Monoclinic, *P*2_1_/*c*
Temperature (K)	296
*a*, *b*, *c* (Å)	15.8644 (5), 7.2242 (2), 12.7595 (4)
β (°)	97.038 (2)
*V* (Å^3^)	1451.32 (8)
*Z*	4
Radiation type	Mo *K*α
μ (mm^−1^)	0.47
Crystal size (mm)	0.34 × 0.23 × 0.14

Data collection	
Diffractometer	Bruker APEXII CCD
Absorption correction	Multi-scan (*SADABS*; Bruker, 2003 ▸)
*T*_min_, *T*_max_	0.861, 0.925
No. of measured, independent and observed [*I* > 2σ(*I*)] reflections	11383, 2851, 2359
*R*_int_	0.019
(sin θ/λ)_max_ (Å^−1^)	0.618

Refinement	
*R*[*F* ^2^ > 2σ(*F* ^2^)], *wR*(*F* ^2^), *S*	0.031, 0.089, 1.05
No. of reflections	2851
No. of parameters	199
H-atom treatment	H-atom parameters constrained
Δρ_max_, Δρ_min_ (e Å^−3^)	0.18, −0.21

Computer programs: *APEX3* and *SAINT* (Bruker, 2007 ▸), *SHELXT2016* (Sheldrick, 2015*a* ▸), *SHELXL2016* (Sheldrick, 2015*b* ▸), *ORTEP-3 for Windows* (Farrugia, 2012 ▸) and *PLATON* (Spek, 2009 ▸).

## supplementary crystallographic information

### Crystal data

**Table:**

C_14_H_8_Cl_2_FN_3_O_2_	*F*(000) = 688
*M**_r_* = 340.13	*D*_x_ = 1.557 Mg m^−^^3^
Monoclinic, *P*2_1_/*c*	Mo *K*α radiation, λ = 0.71073 Å
*a* = 15.8644 (5) Å	Cell parameters from 4877 reflections
*b* = 7.2242 (2) Å	θ = 3.1–25.9°
*c* = 12.7595 (4) Å	µ = 0.47 mm^−^^1^
β = 97.038 (2)°	*T* = 296 K
*V* = 1451.32 (8) Å^3^	Block, orange
*Z* = 4	0.34 × 0.23 × 0.14 mm

### Data collection

**Table:**

Bruker APEXII CCD diffractometer	2359 reflections with *I* > 2σ(*I*)
φ and ω scans	*R*_int_ = 0.019
Absorption correction: multi-scan (SADABS; Bruker, 2003)	θ_max_ = 26.0°, θ_min_ = 3.2°
*T*_min_ = 0.861, *T*_max_ = 0.925	*h* = −14→19
11383 measured reflections	*k* = −8→8
2851 independent reflections	*l* = −15→15

### Refinement

**Table:**

Refinement on *F*^2^	Primary atom site location: structure-invariant direct methods
Least-squares matrix: full	Secondary atom site location: difference Fourier map
*R*[*F*^2^ > 2σ(*F*^2^)] = 0.031	Hydrogen site location: inferred from neighbouring sites
*wR*(*F*^2^) = 0.089	H-atom parameters constrained
*S* = 1.05	*w* = 1/[σ^2^(*F*_o_^2^) + (0.0427*P*)^2^ + 0.3915*P*] where *P* = (*F*_o_^2^ + 2*F*_c_^2^)/3
2851 reflections	(Δ/σ)_max_ = 0.001
199 parameters	Δρ_max_ = 0.18 e Å^−^^3^
0 restraints	Δρ_min_ = −0.21 e Å^−^^3^

### Special details

**Table:**

Geometry. All esds (except the esd in the dihedral angle between two l.s. planes) are estimated using the full covariance matrix. The cell esds are taken into account individually in the estimation of esds in distances, angles and torsion angles; correlations between esds in cell parameters are only used when they are defined by crystal symmetry. An approximate (isotropic) treatment of cell esds is used for estimating esds involving l.s. planes.

### Fractional atomic coordinates and isotropic or equivalent isotropic displacement parameters (Å^2^)

**Table:**

	*x*	*y*	*z*	*U*_iso_*/*U*_eq_	
Cl1	0.97386 (3)	1.20992 (9)	0.70124 (4)	0.07088 (18)	
Cl2	0.92856 (3)	1.13756 (8)	0.90771 (4)	0.06189 (17)	
F1	0.47733 (7)	0.7105 (2)	1.03511 (10)	0.0765 (4)	
O1	0.68767 (10)	1.0573 (2)	0.24070 (11)	0.0720 (4)	
O2	0.80699 (11)	0.9169 (2)	0.23689 (11)	0.0788 (5)	
N1	0.69628 (8)	0.95452 (19)	0.76270 (10)	0.0402 (3)	
N2	0.76871 (8)	1.00408 (18)	0.80363 (10)	0.0390 (3)	
N3	0.75395 (11)	0.9908 (2)	0.28420 (12)	0.0521 (4)	
C1	0.64239 (9)	0.8985 (2)	0.83834 (12)	0.0365 (3)	
C2	0.66658 (10)	0.8947 (2)	0.94654 (12)	0.0406 (4)	
H2	0.720428	0.934987	0.974163	0.049*	
C3	0.61096 (11)	0.8315 (3)	1.01287 (13)	0.0481 (4)	
H3	0.626481	0.828004	1.085535	0.058*	
C4	0.53205 (11)	0.7736 (3)	0.96946 (14)	0.0488 (4)	
C5	0.50562 (11)	0.7772 (3)	0.86378 (15)	0.0539 (5)	
H5	0.451398	0.737848	0.837092	0.065*	
C6	0.56182 (10)	0.8409 (3)	0.79752 (13)	0.0485 (4)	
H6	0.545439	0.845134	0.725037	0.058*	
C7	0.82440 (9)	1.0572 (2)	0.73073 (12)	0.0379 (3)	
C8	0.89916 (10)	1.1249 (2)	0.77426 (13)	0.0440 (4)	
C9	0.80338 (9)	1.0366 (2)	0.61456 (12)	0.0364 (3)	
C10	0.73747 (10)	1.1361 (2)	0.55905 (13)	0.0426 (4)	
H10	0.704682	1.214863	0.595145	0.051*	
C11	0.72030 (10)	1.1192 (2)	0.45128 (13)	0.0433 (4)	
H11	0.675811	1.184705	0.414101	0.052*	
C12	0.77022 (10)	1.0035 (2)	0.39955 (12)	0.0392 (4)	
C13	0.83517 (11)	0.9005 (2)	0.45209 (13)	0.0456 (4)	
H13	0.867698	0.822187	0.415369	0.055*	
C14	0.85078 (10)	0.9161 (2)	0.55996 (13)	0.0433 (4)	
H14	0.893493	0.845300	0.596958	0.052*	

### Atomic displacement parameters (Å^2^)

**Table:**

	*U*^11^	*U*^22^	*U*^33^	*U*^12^	*U*^13^	*U*^23^
Cl1	0.0507 (3)	0.0932 (4)	0.0709 (3)	−0.0250 (3)	0.0163 (2)	−0.0040 (3)
Cl2	0.0511 (3)	0.0837 (4)	0.0477 (3)	0.0004 (2)	−0.0067 (2)	−0.0095 (2)
F1	0.0605 (7)	0.1118 (10)	0.0615 (7)	−0.0204 (7)	0.0249 (6)	0.0118 (7)
O1	0.0832 (10)	0.0843 (10)	0.0443 (7)	−0.0050 (9)	−0.0089 (7)	0.0071 (7)
O2	0.1061 (12)	0.0883 (11)	0.0464 (8)	0.0037 (9)	0.0273 (8)	−0.0124 (7)
N1	0.0380 (7)	0.0491 (8)	0.0341 (7)	0.0013 (6)	0.0068 (5)	−0.0010 (6)
N2	0.0381 (7)	0.0441 (7)	0.0352 (7)	0.0023 (6)	0.0062 (5)	0.0007 (6)
N3	0.0712 (10)	0.0461 (8)	0.0395 (8)	−0.0162 (8)	0.0090 (8)	0.0005 (7)
C1	0.0369 (8)	0.0395 (8)	0.0337 (7)	0.0030 (6)	0.0077 (6)	−0.0024 (6)
C2	0.0386 (8)	0.0453 (9)	0.0374 (8)	−0.0011 (7)	0.0031 (6)	−0.0021 (7)
C3	0.0510 (10)	0.0597 (11)	0.0342 (8)	−0.0030 (8)	0.0076 (7)	0.0020 (8)
C4	0.0464 (9)	0.0572 (11)	0.0458 (9)	−0.0041 (8)	0.0180 (8)	0.0021 (8)
C5	0.0372 (9)	0.0727 (12)	0.0517 (10)	−0.0091 (8)	0.0056 (8)	−0.0067 (9)
C6	0.0428 (9)	0.0670 (12)	0.0353 (8)	−0.0010 (8)	0.0031 (7)	−0.0046 (8)
C7	0.0361 (8)	0.0395 (8)	0.0384 (8)	0.0044 (6)	0.0065 (6)	0.0013 (7)
C8	0.0382 (8)	0.0491 (9)	0.0446 (9)	0.0023 (7)	0.0048 (7)	−0.0012 (8)
C9	0.0331 (7)	0.0395 (8)	0.0376 (8)	−0.0017 (6)	0.0079 (6)	0.0027 (7)
C10	0.0416 (8)	0.0463 (9)	0.0414 (9)	0.0092 (7)	0.0116 (7)	0.0022 (7)
C11	0.0400 (8)	0.0475 (9)	0.0422 (9)	0.0032 (7)	0.0046 (7)	0.0087 (7)
C12	0.0457 (9)	0.0389 (8)	0.0343 (8)	−0.0099 (7)	0.0093 (7)	0.0016 (7)
C13	0.0455 (9)	0.0467 (9)	0.0469 (9)	0.0038 (8)	0.0154 (7)	−0.0044 (8)
C14	0.0384 (8)	0.0473 (9)	0.0447 (9)	0.0086 (7)	0.0069 (7)	0.0031 (7)

### Geometric parameters (Å, º)

**Table:**

Cl1—C8	1.7088 (17)	C5—C6	1.381 (2)
Cl2—C8	1.7120 (17)	C5—H5	0.9300
F1—C4	1.3569 (19)	C6—H6	0.9300
O1—N3	1.225 (2)	C7—C8	1.339 (2)
O2—N3	1.217 (2)	C7—C9	1.486 (2)
N1—N2	1.2547 (18)	C9—C10	1.389 (2)
N1—C1	1.4242 (19)	C9—C14	1.392 (2)
N2—C7	1.4123 (19)	C10—C11	1.374 (2)
N3—C12	1.466 (2)	C10—H10	0.9300
C1—C6	1.384 (2)	C11—C12	1.375 (2)
C1—C2	1.387 (2)	C11—H11	0.9300
C2—C3	1.373 (2)	C12—C13	1.377 (2)
C2—H2	0.9300	C13—C14	1.373 (2)
C3—C4	1.371 (2)	C13—H13	0.9300
C3—H3	0.9300	C14—H14	0.9300
C4—C5	1.362 (3)		
			
N2—N1—C1	113.22 (12)	C8—C7—C9	121.96 (14)
N1—N2—C7	114.73 (13)	N2—C7—C9	123.20 (13)
O2—N3—O1	123.66 (16)	C7—C8—Cl1	122.91 (13)
O2—N3—C12	118.56 (16)	C7—C8—Cl2	123.47 (13)
O1—N3—C12	117.78 (16)	Cl1—C8—Cl2	113.61 (9)
C6—C1—C2	119.92 (14)	C10—C9—C14	119.18 (14)
C6—C1—N1	115.71 (14)	C10—C9—C7	121.30 (14)
C2—C1—N1	124.35 (14)	C14—C9—C7	119.53 (14)
C3—C2—C1	119.97 (15)	C11—C10—C9	120.58 (15)
C3—C2—H2	120.0	C11—C10—H10	119.7
C1—C2—H2	120.0	C9—C10—H10	119.7
C4—C3—C2	118.44 (15)	C10—C11—C12	118.64 (15)
C4—C3—H3	120.8	C10—C11—H11	120.7
C2—C3—H3	120.8	C12—C11—H11	120.7
F1—C4—C5	118.34 (16)	C11—C12—C13	122.39 (15)
F1—C4—C3	118.34 (16)	C11—C12—N3	118.64 (15)
C5—C4—C3	123.32 (16)	C13—C12—N3	118.96 (15)
C4—C5—C6	117.96 (16)	C14—C13—C12	118.43 (15)
C4—C5—H5	121.0	C14—C13—H13	120.8
C6—C5—H5	121.0	C12—C13—H13	120.8
C5—C6—C1	120.38 (16)	C13—C14—C9	120.72 (15)
C5—C6—H6	119.8	C13—C14—H14	119.6
C1—C6—H6	119.8	C9—C14—H14	119.6
C8—C7—N2	114.83 (14)		
			
C1—N1—N2—C7	178.86 (13)	C8—C7—C9—C10	−116.26 (18)
N2—N1—C1—C6	−179.49 (15)	N2—C7—C9—C10	65.2 (2)
N2—N1—C1—C2	−1.1 (2)	C8—C7—C9—C14	63.4 (2)
C6—C1—C2—C3	0.9 (2)	N2—C7—C9—C14	−115.15 (17)
N1—C1—C2—C3	−177.43 (16)	C14—C9—C10—C11	−1.4 (2)
C1—C2—C3—C4	−0.1 (3)	C7—C9—C10—C11	178.25 (15)
C2—C3—C4—F1	179.75 (16)	C9—C10—C11—C12	−0.7 (2)
C2—C3—C4—C5	−0.6 (3)	C10—C11—C12—C13	1.8 (2)
F1—C4—C5—C6	−179.76 (17)	C10—C11—C12—N3	−177.72 (14)
C3—C4—C5—C6	0.6 (3)	O2—N3—C12—C11	166.51 (16)
C4—C5—C6—C1	0.2 (3)	O1—N3—C12—C11	−12.8 (2)
C2—C1—C6—C5	−0.9 (3)	O2—N3—C12—C13	−13.0 (2)
N1—C1—C6—C5	177.56 (16)	O1—N3—C12—C13	167.66 (16)
N1—N2—C7—C8	174.62 (14)	C11—C12—C13—C14	−0.7 (2)
N1—N2—C7—C9	−6.7 (2)	N3—C12—C13—C14	178.84 (15)
N2—C7—C8—Cl1	−176.19 (11)	C12—C13—C14—C9	−1.6 (2)
N2—C7—C8—Cl2	2.9 (2)	C10—C9—C14—C13	2.6 (2)
C9—C7—C8—Cl2	−175.78 (12)	C7—C9—C14—C13	−177.11 (15)

### Hydrogen-bond geometry (Å, º)

**Table:**

*D*—H···*A*	*D*—H	H···*A*	*D*···*A*	*D*—H···*A*
C10—H10···O1^i^	0.93	2.52	3.369 (2)	152

Symmetry code: (i) *x*, −*y*+5/2, *z*+1/2.
